# Untangling the *Hypogeococcus pungens* species complex (Hemiptera: Pseudococcidae) for Argentina, Australia, and Puerto Rico based on host plant associations and genetic evidence

**DOI:** 10.1371/journal.pone.0220366

**Published:** 2019-07-25

**Authors:** Daniel Poveda-Martínez, María Belén Aguirre, Guillermo Logarzo, Luciano Calderón, Alicia de la Colina, Stephen Hight, Serguei Triapitsyn, Hilda Diaz-Soltero, Esteban Hasson

**Affiliations:** 1 Fundación para el Estudio de Especies Invasivas (FuEDEI), Hurlingham, Buenos Aires, Argentina; 2 Instituto de Ecología Genética y Evolución de Buenos Aires (IEGEBA), Departamento de Ecología Genética y Evolución, Facultad de Ciencias Exactas y Naturales, Universidad de Buenos Aires, Buenos Aires, Argentina; 3 Consejo Nacional de Investigaciones Científicas y Técnicas (CONICET), Ciudad Autónoma de Buenos Aires, Argentina; 4 Grupo de investigación en Evolución, Ecología y Conservación (EECO), Universidad del Quindío, Armenia, Colombia; 5 U.S. Department of Agriculture-ARS, Tallahassee, Florida, United States of America; 6 Department of Entomology, University of California, Riverside, California, United States of America; 7 Caribbean Advisor to the APHIS Administrator, USDA, San Juan, Puerto Rico; National Cheng Kung University, TAIWAN

## Abstract

*Hypogeococcus pungens*, a mealybug native of southern South America, is devastating native cacti in Puerto Rico and threatening cactus diversity in the Caribbean, and potentially in Central and North America. The taxonomic status of *H*. *pungens* is controversial since it has been reported feeding not only on Cactaceae but also on other plant families throughout its distribution range. However, in Australia, where the species had been exported from Argentina to control weedy American cacti, it was never found on host plants other than Cactaceae. These conflicting pieces of evidence not only cast doubt on the species identity that invaded Puerto Rico, but also have a negative impact on the search for natural enemies to be used in biological control programs against this pest. Here we present reproductive incompatibility and phylogenetic evidences that give support to the hypothesis that *H*. *pungens* is a species complex in which divergence appears to be driven by the host plants. The nuclear *EF1α* and *18S* and the mitochondrial *COI* genes were used as markers to evaluate the phylogenetic relationships among *H*. *pungens* populations collected in Argentina, Australia and Puerto Rico feeding on Cactaceae and/or Amaranthaceae. Additionally, we conducted reciprocal crosses between mealybugs from both hosts. Species delimitation analysis revealed two well-supported putative species within *H*. *pungens*, one including mealybugs feeding on Amaranthaceae (*H*. *pungens sensu stricto*), and a new undescribed species using Cactaceae as hosts. Additionally, we found asymmetric reproductive incompatibility between these putative species suggesting recent reproductive isolation. The Bayesian species delimitation also suggested that the Australian mealybug population may derive from another undescribed species. Overall, the patterns of genetic differentiation may be interpreted as the result of recent speciation events prompted by host plant shifts. Finally, the finding of a single haplotype in the Puerto Rico population suggests only one invasive event. We still need to identify the geographical origin of the pest in order to enable the use of biological control to reduce the threat to cacti diversity in the Caribbean.

## Introduction

The mealybug *Hypogeococcus pungens* Granara de Willink (Hemiptera: Pseudococcidae), commonly called the *Harrisia* cactus mealybug (HCM), is devastating the native columnar, globular and semi-epiphytic cactus species of Puerto Rico, and threatening cacti throughout the Caribbean, and Central and North America including Mexico [1,2]. *H*. *pungens*, one of the members of the genus *Hypogeococcus* Rau which is native to the New World, was first recorded on cacti in Puerto Rico in 2005; by 2010 the insect had covered an area of 1400 km^2^ and is continuing to spread on the main island [3]. Taking into account that *H*. *pungens* is found at low densities in its native range where its natural enemies are present [4], biological control is the most appropriate management option to protect wild populations of cacti from this mealybug in large natural areas. Chemical control, however, may be feasible for small areas like nurseries and commercial cultivations [5].

*H*. *pungens* is native to South America, mainly Argentina, Bolivia, Brazil, Paraguay, Peru, and Uruguay [5]. The mealybug was originally described from collections on *Alternanthera pungens* Kunth (Amaranthaceae) in Argentina [6]. Outside of its native range, the mealybug has been reported in Australia, South Africa, United States of America, several European countries, and in the Caribbean [7–16]. The Australian and South African *Hypogeococcus* populations were deliberately introduced from Argentina for the biological control of several weedy cacti [15,17–18]. The host range of *H*. *pungens sensu lato* is constrained to members of the Amaranthaceae, Cactaceae, Portulacaceae and Euphorbiaceae [5, 19–20].

However, a word of caution is needed regarding the accuracy of insect-host-plant associations and geographical distribution because there is a certain degree of confusion about the taxonomic status of *H*. *pungens*. *H*. *pungens* has been misidentified as *Hypogeococcus festerianus* Lizer & Trelles (a valid species restricted to Cactaceae) in several publications [8, 21–25]. McFadyen & Tomley [22,23] and Tomley & McFadyen [8] reported the successful biological control of invasive cactus with the “Harrisia cactus mealybug” then identified as *H*. *festerianus* [18]. However, the identification of this mealybug was conducted by Williams [4] before the description of *H*. *pungens*. In fact, the species introduced into Australia was subsequently identified as *H*. *pungens* by Williams & Granara de Willink [1]. The paradox is that McFadyen [8] reported that the species introduced into Australia was only found in the field on Cactaceae host plants, in spite of the availability of members of Amaranthaceae and Portulacaceae families that are common in the wild. In addition, the *Hypogeococcus* species introduced into Australia did not develop in any of these families in the laboratory. In biological control publications, Australian researchers continue using *H*. *festerianus* instead of *H*. *pungens* because of the polyphagous behavior of the latter [8].

A recent study evaluating aspects of the biology of *H*. *pungens* revealed marked differences between mealybugs collected on Amaranthaceae (hereafter *H*. *pungens sensu stricto*) and on Cactaceae, giving strong support to the idea that *H*. *pungens* is, in fact, a species complex [26]. Even though the progeny of gravid *H*. *pungens* collected on Amaranthaceae in nature was able to complete development on cactus hosts, it could not produce a viable second generation on Cactaceae [26]. Moreover, differences in fecundity, female body length, mode of reproduction, rate of development, and host range were also observed between *H*. *pungens sensu stricto* and the mealybugs introduced into Australia [4]. In this sense, it is worth noting that *H*. *pungens sensu stricto* can reproduce by both sexual reproduction and parthenogenesis by deuterotoky, whereas cactus breeding bugs only reproduce sexually [26].

In 2010, as part of a biological control program against this cactus pest in Puerto Rico (USDA, ARS 2014), the Fundación para el Estudio de Especies Invasivas (FuEDEI) started surveying for *H*. *pungens sensu lato* in Argentina on host plants of *Alternanthera* spp., other Amaranthaceae species, and species of native cacti. The survey had two goals, to search for the most promising biological control agents, and to identify the population of *Hypogeococcus* spp. that matched the Puerto Rico cactus pest. Surveys began in Argentina because *H*. *pungens* was originally described from specimens collected in this country. Also, Argentina was the country with the most known *Hypogeococcus* mealybug species that infested Cactaceae: *H*. *pungens as well as H*. *festerianus* and *Hypogeococcus spinosus* Ferris both restricted to Cactaceae.

Finally, although Zimmermann et al. [5] suggested that the most likely dispersion of *Hypogeococcus* was via nursery trade and wind dispersal, we could not rule out that HCM populations of Puerto Rico arrived from Australia or South Africa, where “*H*. *pungens*” specimens had been introduced from Argentina.

In classical biological control programs, the correct identification of the target species and its natural enemies is a key issue [27]. The kind of biological control selected to manage the Puerto Rico pest strongly depends on the precise identification of the mealybug species pest. Here we address three basic questions using molecular markers and reciprocal cross-breeding experiments: 1) Is *H*. *pungens* a complex of closely related species, that would explain the differences observed in the host-range breadth? 2) Is the identity of the mealybug introduced into Australia for control of cactus weeds *H*. *pungens*, *H*. *festerianus*, or a new undescribed species? and finally, 3) Which is the source population of the Puerto Rico mealybug cactus pest?

## Materials and methods

### Samples analyzed

**Fig 1:**
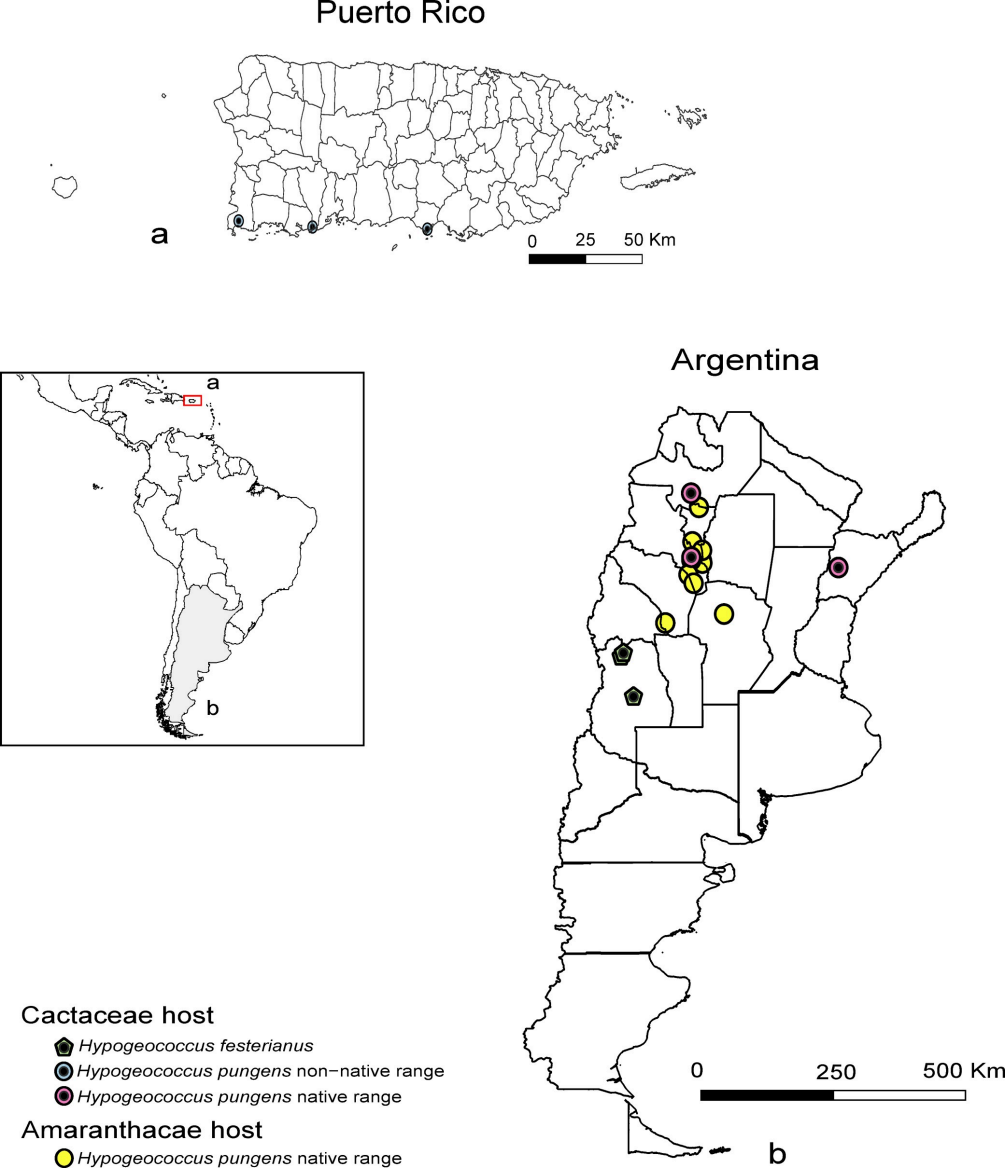
Geographical distribution of *Hypogeococcus pungens sensu lato* samples used in the genetic study. The map shows the locations sampled from the native (Argentina) and non-native (Puerto Rico) range of the mealybug *H*. *pungens sensu lato*, and the co-generic species *H*. *festerianus*.

Field surveys of *Hypogeococcus* populations, considered to be *H*. *pungens*, were conducted in Argentina, Australia and Puerto Rico between 2010 and 2016. Our study did not involve any endangered or protected insect species. For the mealybugs collected from Argentine, we obtained the permission from the land owners and no specific permits were required in Australia. However, for the mealybugs collected from Puerto Rico, we obtained the permission from the U.S Fish & Wildlife Service (Permit No. 41522-16-003), and Department of Natural and Environmental Resources (Permit No. OV-1617-15). Mealybugs were collected on Amaranthaceae and/or Cactaceae, following the host range reported for *H*. *pungens* [1, 19–20]. 10–50 plants of each potential host were inspected for mealybugs. If mealybugs were present, 5–20 adult specimens (depending on abundance) were preserved in absolute ethanol for genetic survey. Our study encompassed sites across 14 Argentine provinces (Salta, Jujuy, Tucumán, La Rioja, Catamarca, Córdoba, Mendoza, Santiago del Estero, Formosa, Chaco, San Juan, San Luis, Entre Ríos, and Corrientes). Since *H*. *pungens* was misidentified as *H*. *festerianus* in the past, we included 10 *H*. *festerianus* females collected on *Cereus aethiops* Haworth (Cactaceae) in Mendoza Province, as well as 10 specimens collected on cacti in Australia whose taxonomic status is still unclear [8]. Fifteen adult females, collected in Puerto Rico and tentatively identified as HCM (5 ♀ from Cabo Rojo Municipality, 5 ♀ from Guánica Municipality, and 5 ♀ from Caja de Muertos Island, Ponce Municipality) were included in the study with the aim of identifying the source populations of the cactus pest (S1 Table). The vine mealybug, *Planococcus ficus* (Signoret) (Hemiptera: Pseudococcidae), was used as an outgroup in the phylogenetic studies. S1 Table shows the geographic coordinates of each sampling site along with other relevant information (host plant family, host plant species, and sample size) (Fig 1).

### DNA extraction, PCR amplification and sequencing

Genomic DNA extractions were performed using the whole female body with the Qiagen DNeasy Blood & Tissue Kit following manufacturer’s instructions (Qiagen, Inc., Valencia, California, USA). For the samples collected in Argentina and Australia, we obtained information on three different genes, the mitochondrial Cytochrome Oxidase subunit I (*COI*), and two nuclear genes: translation elongation factor (*EF1α*) and *18S* Ribosomal RNA. For the samples from Puerto Rico, we only obtained the *COI* gene because it was much more informative than the other markers. The RNA *18S* gene was amplified using primers 18S-2880 (5’-CTGGTTGATCCTGCCAGTAG-3) and 18S-B (5’-CCGCGGCTGCTGGCACCAGA-3’) [28]; *EF1α* using primers M51.6 (5’-CARGACGTATACAAAATCGG-3’) and rcM53-2 (5’-CAATGTGRGCIGTGTGGCA-3’) [29]; and *COI* with primers C1-J-2183 Jerry (5’-CAACATTTATTTTGATTTTTTGG-3’) [30] and C1-N-2568 BEN3R (5’-GCWACWACRTAATAKGTATCATG-3’) [31]. The PCR reaction components for all amplifications were 12.5 μL of Taq polymerase in the appropriate buffer (PCR Master Mix-Promega, Madison, WI, USA), 1 μL of each primer [5 mM], 1 μL of DNA template and 12.5 μL of nuclease free water to complete a total volume of 25 μL. The PCR cycling protocol for *COI* and *18S* was as follows: an initial denaturing step at 98°C for 1 min, followed by 35 cycles of 98°C for 30 sec, 56°C for 40 sec, and 72°C for 1 min, with a final extension at 72°C for 10 min. For *EF1α* the PCR protocol was 94°C for 4 min, followed by 35 cycles of 94°C for 1 min, 49°C for 1 min, and 72°C for 1.5 min, with a final extension at 72°C for 4 min. PCR products were checked on a 1% agarose gel, and purified using the QIAquick PCR Purification Kit (Qiagen, Inc., Valencia, California, USA). Finally, both strands of each fragment were sequenced using Sanger technology in Macrogen Inc. sequencing service (Seoul, South Korea). All sequences were used in *blastn* searches to discard sequences belonging to mealybug parasitoids [32].

### Data analysis

Both strands of all sequences were assembled into contigs using CodonCode Aligner v8.0.2 (CodonCode Corporation, Centerville, Massachusetts, USA), and multiple sequence alignment for each gene was performed using ClustalW algorithm in MEGA v. 7 [33]. The saturation substitution index (Iss) was estimated for each gene to evaluate if the Iss was significantly lower than the critical value of Iss (Iss.c). This test is necessary to ascertain whether sequences are phylogenetically informative [34]. Iss and the proportions of conserved and polymorphic sites were computed using DAMBE v.5.5.1 [35].

### Phylogenetic analyses

We explored the relationships among *H*. *pungens sensu lato* populations and the relationship between *H*. *pungens* and its co-generic *H*. *festerianus* by means of a phylogenetic analysis using Bayesian inference (BI) and Maximum Likelihood approaches. In both cases, we utilized a concatenated data set using a partition scheme with three blocks (*18S*, *EF1α* and *COI*) as input. To exclude redundancies, the data set was trimmed using a single individual per haplotype. The General Time Reversible + Gamma parameter (*GTR+Γ*) model was adopted for each partition, as determined by means of Bayesian information criterion as suggested in Jmodeltest v.2.1.3 [36]. The related mealybug *Planococcus ficus* was used as outgroup. The BI was implemented in MrBayes v3.2.6 [37] and the ML analysis in RAxML-HPC2 v.8.2.10 [38] in CIPRES [39]. One thousand bootstrap replicates were run to evaluate clade stability. Two independent runs were performed with four Markov Chain Monte Carlo (MCMC) chains for each run for 50 million generations, sampling every 1000^th^ generation. The first 25% of samples were removed as burn-in, and stability and sufficient mixing of parameters (ESS>200) was checked using Tracer v.1.6 [40]. Independent ML and BI analyses of each gene were performed and are available in supplementary material (S1 Fig). Finally, we estimated genetic divergence using uncorrected p-distances and the average number of pairwise differences between and within clades obtained in the phylogenetic analysis, using *COI* sequences, and including the Puerto Rico cactus pest.

### Species delimitation analyses and divergence time

To evaluate whether *H*. *pungens* represented a unique species or a species complex, we used two types of species delimitation analyses: i) a multi-locus Bayesian approach [41] and ii) two single-locus approaches: Automatic Barcode Gap Discovery (ABGD) distance-based method [42] and multi-rate Poisson tree processes (mPTP) ML tree-based method [43]. In the multi-locus Bayesian analysis, we considered the well-supported clades identified in the phylogenetic analyses, combined with the results of experimental studies showing potential postzygotic reproductive barriers between mealybugs collected on Amaranthaceae and Cactaceae from Argentina, and the incapability of specimens collected on Cactaceae to develop and survive on Amaranthaceae and *vice versa* [26]. We employed the set of species trees estimated with BEAST v1.7.5 [44], including *H*. *pungens*, *H*. *festerianus* and the outgroup *P*. *ficus*, using the sequences of the three genes. This multi-locus method is based on incomplete lineage sorting as the main source of inconsistency between gene trees and species trees and assumed free recombination between genes and no recombination within genes. In this case, terminal taxa were defined *a priori* considering two alternative hypotheses. The first (hypothesis A) considered *H*. *pungens* as a complex of two species, one consisting of populations feeding on Amaranthaceae and the other of populations using Cactaceae. In the second scenario (hypothesis B), *H*. *pungens* is a complex of three species, namely, *H*. *pungens sensu stricto* including Amaranthaceae dwelling populations, and two new undescribed species both specialized in the use of Cactaceae: *Hypogeococcus* sp. 1 and *Hypogeococcus* sp. 2 encompassing the populations from Argentina and the specimens collected in Australia, respectively. Both hypotheses were tested by means of a Bayesian Markov Chain Monte Carlo analysis running 50 million generations and sampled every 1000th generations with a Yule Process as a tree prior and a strict clock model estimated according to *COI* partition. Convergence was verified with Tracer 1.6 [40] and the first 20% of sampled trees were discarded as burn-in using TreeAnnotator v1.7.5 [44]. Species trees resulting from the Bayesian analysis were visualized on DensiTree v2.1 [45]. We estimated divergence time between the entities identified by the species delimitation analyses, using a substitution rate of 0.017 substitutions per site per million years estimated for insects [46]. A posterior probability value ≥ 0.95 was considered as strong support for a speciation event [47].

In the single-locus analyses, ABGD and mPTP, we used the mitochondrial *COI* gene from all populations sampled in Argentina and Puerto Rico. We included the latter to determine which of the groups identified using these approaches was more related to the Puerto Rico cactus pest. The ABGD analysis was run in the ABGD website server (http://wwwabi.snv.jussieu.fr/public/abgd/abgdweb.html). This analysis uses the barcode gap, which is the gap observed when divergence among individuals of the same species is smaller than divergence among individuals from different species, to automatically find groups that might correspond to different potential species [42,48]. The range of prior intraspecific divergence (P), estimated with uncorrelated p-distances, was configured from 0.001 to 0.1 in 10 Steps, using a gap width of 1.5. [49–50]. The mTPT analysis was performed in mptp v0.2.3 and takes into account the distinct values of intraspecific divergence caused by the substitutions that should confer more credibility in the speciation events [43]. This method assumes that the number of substitutions between species is significantly higher than within species [51]. A ML tree reconstructed with RAxML using the same parameters used in the phylogenetic analysis (substitution rate model, number of bootstrap replicates, and outgroup) was used as input. Identical sequences were removed to avoid incorrect likelihood estimations. The support of the delimited species was assessed using two independent MCMC chains for 10 million generations each, discarding the first 25% as burn-in.

### Reproductive compatibility

We evaluated reproductive compatibility between samples collected on the two main clades identified in phylogenetic analyses (see Results section for details). To this end, we conducted both reciprocal crosses between mealybugs collected in Amaranthaceae (*Alternanthera pungens;* Trancas, Tucumán province) and Cactaceae (*Cleistocactus baumannii;* El Portezuelo, Catamarca province) as follows: (♀Amaranthaceae host x ♂Cactaceae host) and (♀Cactaceae host x ♂Amaranthaceae host) and the respective controls (♀Amaranthaceae host x ♂ Amaranthaceae host) and (♀Cactaceae host x ♂ Cactaceae host). The response variable measured was the number of nymphs that reached adulthood per female (viable offspring). The absence of viable offspring in the crosses was assumed as reproductive incompatibility between mealybugs from different hosts. Since females of the Amaranthaceae clade can reproduce by parthenogenesis [26], we used offspring sex ratio as an indicator of parthenogenetic reproduction. A Chi-square (*χ*^*2*^) test was used to determine if the sex ratio deviated from 1:1 in the offspring (similar number of males and females). On the other hand, we considered that nymphs were sexually produced when sperm was observed in females spermathecae. To this end, ten espermathecae were inspected in Amaranthaceae clade females (five control and five treatment), and ten in Cactaceae clade females (five control and five in treatment).

## Results

### Sequence analysis

A total of 116 individuals were sampled for the present study from which a total of 304 sequences were generated. The basic statistics of sequence variation (number of segregating sites, number of haplotypes) and number of sequences per gene are detailed in S2 Table. A concatenated dataset of 1415 bp was used in the analyses described below. Excluding the gaps between sequences, we obtained 1393 bp of which 1247 bp (89.51%) were conserved positions and 146 bp (10.48%) were polymorphic (all sequences were deposited in GenBank under accession numbers MN013440—MN013743). The saturation substitution analysis revealed no saturation of phylogenetic signal in the set of sequences generated, according to the comparison between the Iss and the Iss critical (Iss.c) (S2 Table).

### Phylogenetic analyses

**Fig 2:**
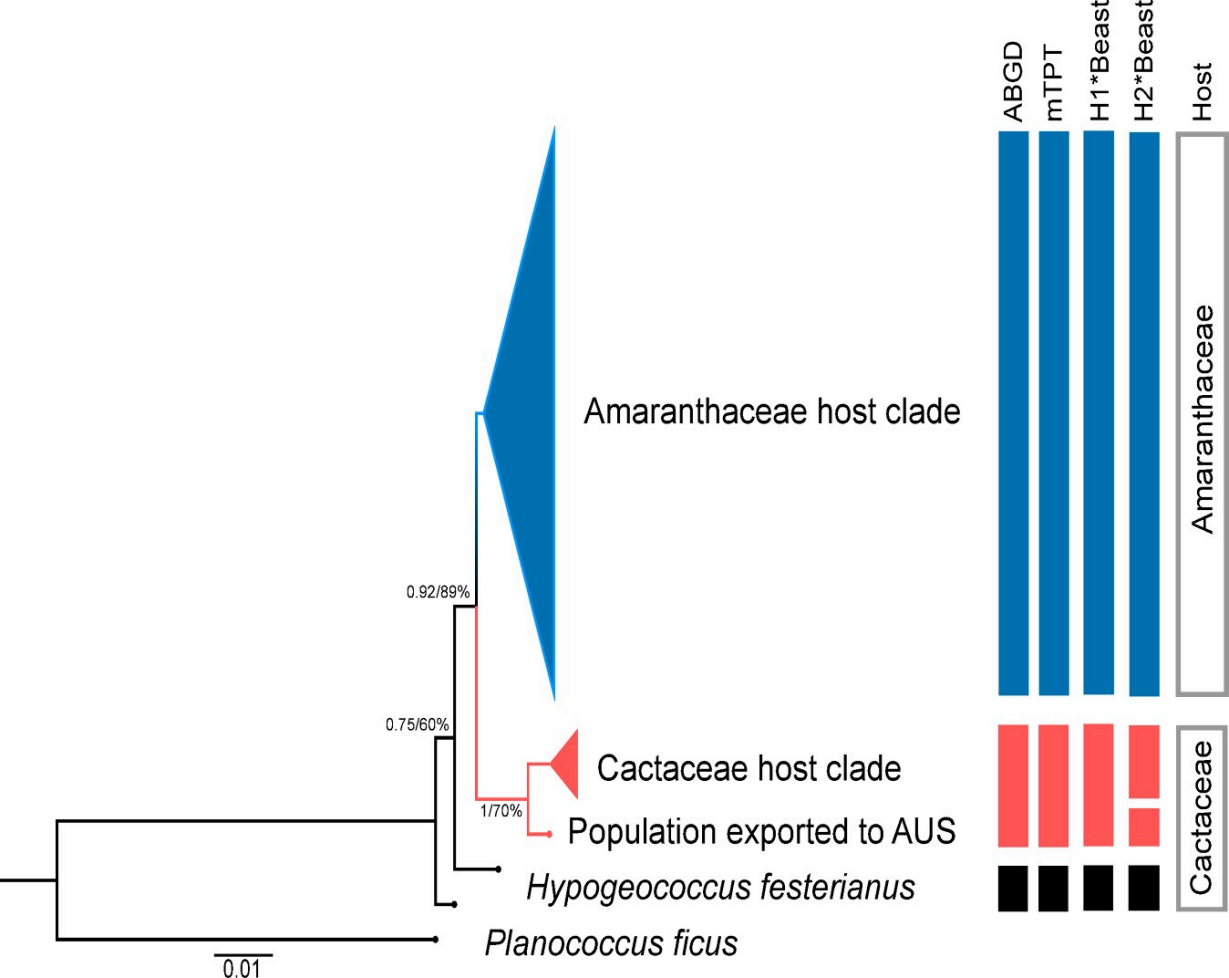
Phylogenetic reconstruction by means of Bayesian inference and maximum likelihood and species delimitation results. Posterior Probability and Bootstrap Support values are shown on each node inferred by BI and ML in a concatenated data set of *COI*, *18S* and *EF1*. Both results are taken to represent the phylogenetic reconstruction of the populations of *H*. *pungens* using different hosts and the co-generic species *H*. *festerianus*. The vertical bars represent the candidate species inferred by each of the methods used in the study (see below).

Two main clades were observed in the BI and ML trees with high Posterior Probability (PP = 0.92) and Bootstrap Support (BS = 89%) (Fig 2) using the concatenated dataset (independent BI and ML trees for each gene are available in S1 Fig). The first clade includes all haplotypes of mealybugs feeding on Amaranthaceae (*Amaranthaceae host clade*), while the second clade contained all haplotypes of individuals collected on cactus hosts (*Cactaceae host clade)*. The unique haplotype found in the Australian samples appears as the sister group of the *Cactaceae host clade* with high PP (1) and BS (70%). As for *H*. *festerianus*, we recovered two different haplotypes that formed independent clades with high PP (0.75) and intermediate BS (60%) (Fig 2). The results from the bayesian and ML analyses with each separate gene were moderately concordant with the results obtained with the concatenated data set (S1 Fig).

### Species delimitation analyses

Pairwise genetic differences between clades defined in the ML tree, measured with uncorrected p-distances, ranged from 1.4% to 3.7%, while differentiation within clades varied from 0% in Australian Population and the Puerto Rico cacti pest, to 0.67% in the *Amaranthaceae host clade*. The barcode gap assumes that divergence among individuals of the same species should be smaller than between species (Table 1). Our results showed that divergence between the *Amaranthaceae* and *Cactaceae host clades* was 3.0%, a value that is about five times greater than within clades (Table 1). Also, divergence estimates between either the *Amaranthaceae host clade* (3.2%) or the *Cactaceae host clade* (1.4%) with Australian mealybugs were also greater than estimates within clades. In turn, genetic divergence between *H*. *festerianus* and the *Amaranthaceae host clade* was 2.1%; whereas divergence between the *Cactaceae host clade* and *H*. *festerianus* was 3.4%. Finally, divergence estimates between the Puerto Rico cactus pest and the *Cactaceae host clade* and the *Amaranthaceae host clade*, both considered as *H*. *pungens*, was 3.5% and 1.6%, respectively (Table 1).

**Table 1:** Uncorrelated p-distances (lower) and average number of differences (upper) between and within clades and populations.

Populations / clade	Amaranthaceae host clade	Cactaceae host clade	Australia Population	*H*. *festerianus*	Puerto Rico cacti pest	p-distance within Clades
**Amaranthaceae host clade**	-	10.762	12.038	7.909	6.189	0.006
**Cactaceae host clade**	0.030	-	5.160	12.880	13.160	0.005
**Australian Population**	0.032	0.014	-	14.000	14.000	0
***H*. *festerianus***	0.021	0.034	0.037	-	10.000	0.003
**Puerto Rico cacti pest**	0.016	0.035	0.037	0.027	-	0

The three methods of species delimitation, the ABGD, the mTPT, and the Bayesian analyses, suggested that *H*. *pungens* was, in fact, a species complex. On one hand, the ABGD and the mTPT approaches detected two candidate species within *H*. *pungens*. The first, not only included populations grouped in the *Amaranthaceae host clade*, but also the Puerto Rico cactus pest. The second species candidate contained the *Cactaceae host clade*. Finally, *H*. *festerianus* formed an independent group in both ABGD and mTPT analyses (S2 and S3 Figs).

Furthermore, the results of the Bayesian analysis gave support to both hypotheses (A and B) of speciation events. Under Hypothesis A, consistent with the results obtained with the ABGD and the mTPT analyses, *H*. *pungens* was a complex that included at least two species: *H*. *pungens sensu stricto* (the *Amaranthaceae host clade*) and *Hypogeococcus* sp. 1 (the *Cactaceae host clade*) (Posterior Probability = 0.995; S4 Fig). The time calibrated analysis suggested that the speciation event separating these inferred species occurred nearly 35,000 years ago (confidence interval 95% HDP = 13,200–88,000), and that both shared their last common ancestor with *H*. *festerianus* approximately 65,000 years ago (confidence interval 95% HDP = 30,800–116,600). The Bayesian analysis also gave support to Hypotheses B (PP > 0.95), indicating that *H*. *pungens* was a complex of three species. The first split occurred nearly 29,000 years ago (confidence interval 95% HDP = 10,912–49,104) separating the *Amaranthaceae* and the *Cactaceae host clades* (PP = 1). The second speciation event took place approximately 10,000 years ago (confidence interval 95% HDP = 2,081–16,368) and separated two species among cactus feeding mealybugs, *Hypogeococcus* sp. 1, encompassing the populations collected in Argentina (*Cactaceae host clade* in Fig 2) and *Hypogeococcus* sp. 2 involving the Australian samples (PP = 0.95) (Fig 2 and S4 Fig). Finally, *H*. *festerianus* appeared to be closely related to the *H*. *pungens* species complex, from which it diverged about 44,000 years ago (confidence interval 95% HDP = 21,824–70,928). For both hypotheses, the total of 37,500 trees generated in Bayesian analyses before burn-in and plotted in DensiTree showed congruent topologies (S4 Fig).

### Reproductive compatibility

The results of reproductive compatibility experiments suggested asymmetrical incompatibility. Control crosses of mealybugs from both hosts produced viable offspring (71.2% of 208 nymphs and 81.1% of 164 nymphs of the Amaranthaceae and Cactaceae host clades, respectively, reached the adult stage). In the inter-clade reciprocal crosses, 65.4% of 191 nymphs produced by Amaranthaceae clade females crossed with Cactaceae clade males reached adulthood (Table 2). In contrast, none of the 84 nymphs produced by Cactaceae clade females crossed with Amaranthaceae males reached the adult stage. In fact, none of the offspring produced in the latter cross developed beyond the second instar.

**Table 2:** Summary of the results of the reciprocal crosses between mealybugs from Amaranthaceae and Cactaceae host (N. Rep = number of replicates).

Crosses	N. Rep.	Successful crosses	Viable offspring (%)	Sex ratio (♀:♂)
**♀Amaranthaceae x ♂Cactaceae**	5	5	65,4	1.7:1 (*χ*^*2*^ = 8.57, *df* = 1, *P* = 0.003)
**♀Cactaceae x ♂Amaranthaceae**	5	0	0,0	-
**♀Amaranthaceae x ♂Amaranthaceae**	5	5	71,2	2:1 (*χ*^*2*^ = 14.3, *df* = 1, *P* < 0.001)
**♀Cactaceae x ♂Cactaceae**	5	5	81,1	1.2:1 (*χ*^*2*^ = 1.27, *df* = 1, *P >* 0.05)

When females were dissected, we detected the presence of sperm in different proportions between treatments and controls (Table 2). In the hybrid crosses, females from Amaranthaceae host x males from Cactaceae host, we found sperm in the spermathecae in 3/5 dissected females; while in the control (♀Amaranthaceae x ♂Amaranthaceae) we observed that 4/5 females presented sperm in their spermathecae. In the reciprocal crosses, females from Cactaceae host x males from Amaranthaceae host, we found sperm in only 1 out of 5 females analyzed, while in Cactaceae host controls, 3/5 females had sperm in their spermathecae.

In both treatment and control crosses involving Amarathaceae host females we detected a biased sex-ratio. Sex ratio in the controls was 2:1 **♀:♂** (range 1:1–4:1 **♀:♂**) and in the female from Amaranthaceae host x male from Cactaceae host, crosses were 1.7:1 **♀**:**♂** (range 1:1–6:0 **♀:♂**). In contrast, we did not detect departures from the expected 1:1 sex ratio in crosses involving females from Cactaceae host (range 1:1–2:1 ♀:♂).

## Discussion

The results of the present study indicated that mealybugs feeding on Amaranthaceae and Cactaceae collected in Argentina, on Cactaceae in Australia, and the Puerto Rico cactus pest, all referred to as *H*. *pungens* in the literature, are part of a species complex. Moreover, the genetic divergence in the *H*. *pungens* complex seemed to be strongly driven by the host plants. In this sense, *H*. *pungens sensu stricto* (the *Amaranthaceae host clade* in Fig 2, and *H*. *pungens* in S2–S4 Figs) appeared as a well-delimited species associated exclusively with the plants of the Amaranthaceae family in Argentina. However, the Puerto Rico cactus pest also appeared as part of the *Amaranthaceae host clade*, though genetically differentiated from all the populations from Argentina feeding on Amaranthaceae. The other members of the complex, which appeared to be ancestral of the mealybugs feeding on Amaranthaceae hosts, were specifically associated with Cactaceae (*Cactaceae host clade* in Fig 2, S2–S4 Figs).

Thus, we may ask whether the degree of genetic divergence among the clades feeding on different host plants could be interpreted as the result of speciation prompted by ecological interactions between mealybugs and their hosts. Such interactions might be an essential factor facilitating ecological speciation. Both types of hosts, Cactaceae and Amaranthaceae, coexist in some localities of northern Argentina (Fig 1), suggesting that feeding on alternative host plants was not only an effective isolating barrier, but also that specialization to particular hosts may have contributed to diversification in *Hypogeococcus* mealybugs.

The results of species delimitation analyses, using alternative algorithms implemented in different software (ABGD, mTPT, and *Beast), all revealed that *H*. *pungens* was a species complex of, at least, two species strongly associated to different host types. The literature pointing to a role of the use of alternative host plants in the rapid diversification in phytophagous insects is vast [52–55]. In this context, recent studies using molecular markers uncovered several cases of host associated genetic structure in plant feeding insects and invoked a role of host shifts in the evolution of reproductive barriers in the initial stages of speciation [52, 56–57]. For instance, studies in the phytophagous ladybird beetle, *Henosepilachna diekei* Jadwiszczak & Węgrzynowicz (Coleoptera: Coccinellidae), show that individuals collected in nature on distinct host plants not only exhibit extremely different host preferences and host performances, but also substantial genetic divergence [58,59]. Similarly, Ebel et al. [54] reports that the host use identification of an ecologically important relationship of the Neotropical *Adelpha* spp. (Lepidoptera: Nymphalidae) butterflies with a toxic host plant family, promotes the rapid diversification of the genus. Similarly, recent studies suggest the central role that host plant shifts play in the diversification of the gall midge genus *Asphondylia* Loew (Diptera: Cecidomyiidae) [60]. All in all, these examples suggest that the evolution of new adaptations to alternative host plants, as the result of divergent selection in alternative hosts, is the main cause of niche expansion and in the emergence of new insect species [52].

Even though there is no strict consensus on the degree of genetic divergence necessary to delimit species [61–64], the extent of genetic divergence among species estimated in our study (Table 1) was consistent with the results obtained in several orders of phytophagous insects in which cryptic species were disclosed using genetic markers [65,66]. Genetic distances among the species uncovered in our study, ranging from 1.4% to 3.7%, were consistent with values reported in other groups in which molecular approaches allowed the delimitation of new species in Hemiptera. Lee et al. [67], for instance, reported distance values greater than 2% in 1595 out of 1694 pairwise comparisons between aphid species. Other authors propose divergence values of up to 3.5% to delimit species in Hemiptera [62,68]. This limit has been calculated based on the highest intra- and interspecific genetic distances in the species complex of *Bemisia tabaci* (Gennadius) (Hemiptera: Aleyrodidae) [62,64,69]. However, such limit is not applicable to recent speciation events, since intra and, specially, interspecific genetic variation is frequently low [70–72], as seems to be the case in the *H*. *pungens* complex. It has been shown in other mealybugs that genetic divergence between congeneric species ranges from 2% to 19.5% [70,72]. In addition, Daane et al. [73] reported divergence estimates, using *COI* as genetic marker, between the sibling species *Planococcus minor* (Maskell) and *P*. *citri* (Risso) of 2%. Divergence values lower than 1% between well-defined species have also been reported in several species of Hemiptera [74–76].

Reproductive compatibility experiments produced evidence supporting the hypothesis that *H*. *pungens* is a species complex and that *Amaranthaceae* and *Cactaceae* host clades are different species. In effect, crosses between specimens collected on Amaranthaceae and Cactaceae revealed asymetrical postzygotic isolation; no F1 offspring could develop beyond the second nymph stage in the cross Cactaceae clade females x Amaranthaceae clade males, whereas similar proportions of F1 offspring reached adulthood in the reciprocal inter-clade crosses as compared to the respective control crosses. Even though we did not evaluate fertility in inter-clade F1 offspring that reached adulthood (Amaranthaceae females x Cactaceae males), our results are consistent with the hypotheses that the clades identified in our genetic survey may be considered different species and that adaptation to new hosts has directly or indirectly driven the evolution of isolating barriers [52,77–78]. In this context, evolutionary studies in *Drosophila* Fallén (Diptera: Drosophilidae) species co-distributed in northwestern Argentina, showed that the differential use of alternative cactus hosts promoted different adaptations (several fitness related traits and patterns of gene expression) that may be the consequence of the association to chemically different hosts [79]. However, testing this hypothesis in *Hypogeococcus* mealybugs and their hosts will require further studies involving a greater representation of the genome and transcriptomic data.

Overall, our genetic analyses indicate that the cactus feeding *Hypogeococcus* sp. mealybug introduced into Australia from Argentina was neither *H*. *pungens sensu stricto* nor *H*. *festerianus*. All species delimitation analyses suggested that mealybugs feeding on cacti, including the population exported to Australia, represent a new species. However, the Bayesian species delimitation analysis supported the hypothesis that the mealybugs introduced into Australia belong to a species that was not sampled in this thorough multi-year survey in its native range. In this context, it is worth mentioning that the Bayesian species delimitation method was more powerful than the distance-based ABGD and ML tree-based mTPT, which only used the mitochondrial gene. This is particularly true in recently formed species groups with low divergence (approximately 10,000 years ago according to the divergence time analysis), since ABGD is prone to false negatives, i.e. lumping less divergent clades in the same candidate species [80]. These results agree with Aguirre et al. [26] who proposed that the population introduced into Australia for biological control of the cactus *Harrisia martinii* (Labouret) Britton is not *H*. *festerianus*, but a new undescribed species. The present paper is expected to encourage future taxonomic work that would provide a formal description of the candidate species unveiled by our population genetic survey, denoted here as *Cactaceae host clade*, as well as new studies involving a wider genome exploration. Studies of reproductive isolation, such as cross-breeding experiments under quarantine conditions between Australian mealybugs and populations derived from *Cactaceae host clade*, are necessary to confirm the species status of the mealybugs exported to Australia. Those tasks are not only relevant but necessary for the design of biological control strategies for pest control in general, and specifically in the use of mealybugs as control agents of weedy cacti.

Finally, the detection of only one haplotype in the three localities sampled of the Puerto Rico cactus pest suggested only one invasive event. This haplotype was not found among the populations sampled in Argentina, the putative origin of the invasive stock. Samples of HCM from Puerto Rico appeared more related to *H*. *pungens* that feed on Amaranthaceae than to the cactus feeding mealybugs from Argentina, including the valid species *H*. *festerianus* and the population from Australia (Table 1). Thus, it seems possible that the Cactaceae feeding condition of the Puerto Rico pest may reflect a very recent host shift. However, identification of the geographic origin of the Puerto Rico cactus pest remains elusive. Identifying the origin of this pest is a mandatory task required to implement an effective plan for biological control to reduce the damage to cactus diversity in Puerto Rico, the adjacent Caribbean islands, and the potential expansion to the neighboring areas in the region. Therefore, new approaches should be implemented using methodologies that allow a more thorough sampling of the genome based on next generation sequencing techniques, including samples from different countries where populations of the *H*. *pungens* complex were reported.

## Supporting information

S1 Fig Maximum Likelihood and Bayesian inference trees of each gene analyzed. (TIF)

S2 Fig Distance-based method Automatic Barcode Gap Discovery (ABGD). (TIF)

S3 Fig ML tree-based method mTPT. (TIF)

S4 Fig Bayesian species delimitation. (TIF)

S1 Table Geographical origin of the individuals analyzed. (DOCX)

S2 Table Substitution saturation index test and sequences variation information. (DOCX)
