# Supplementary figures and images for: Untangling the Hypogeococcus pungens species complex (Hemiptera: Pseudococcidae) for Argentina, Australia, and Puerto Rico based on host plant associations and genetic evidence
Source: PLoS One. 2019 Jul 25;14(7):e0220366. doi: 10.1371/journal.pone.0220366 (PMC6657911; doi:10.1371/journal.pone.0220366)

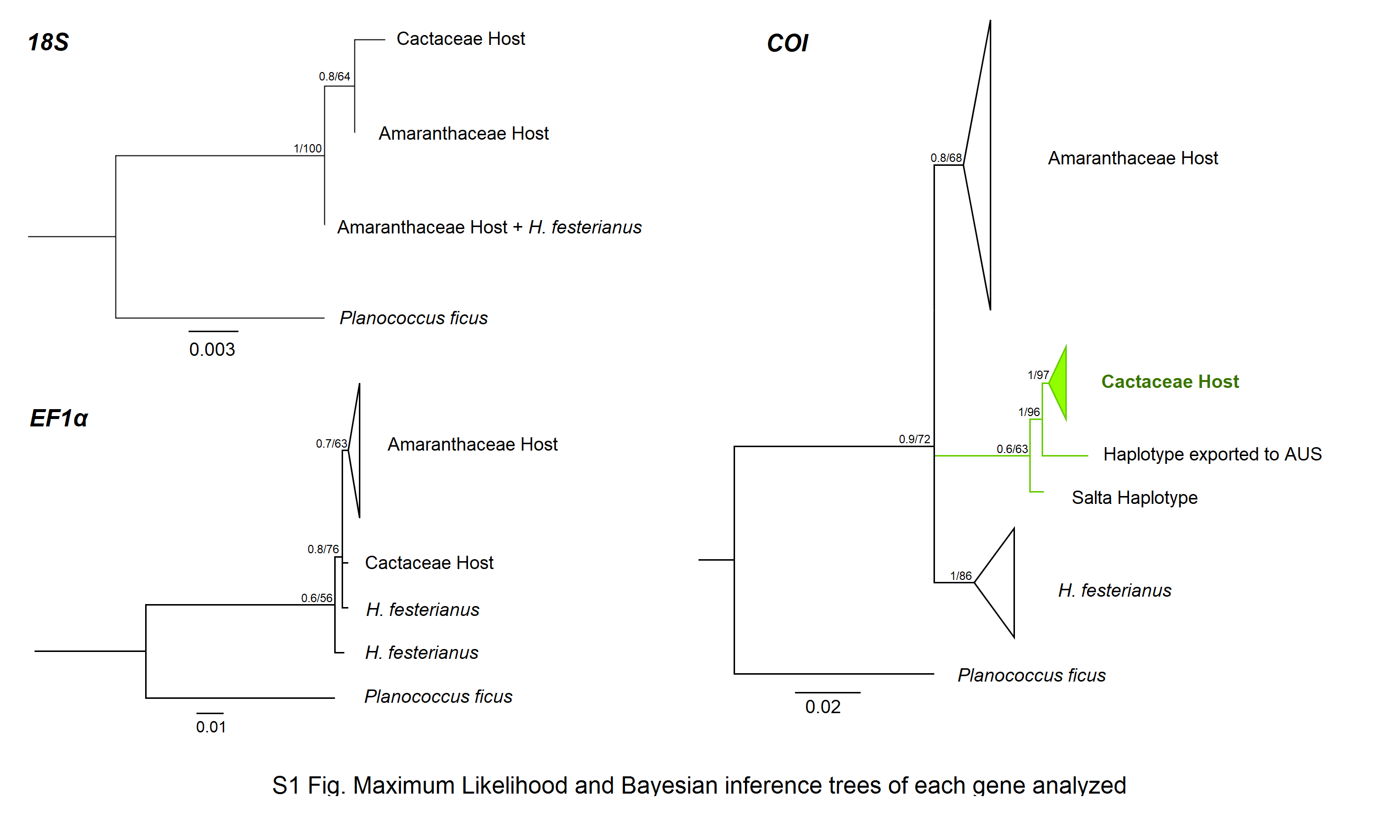

Supplement: S1 Fig — (TIF) [file pone.0220366.s001.tif]

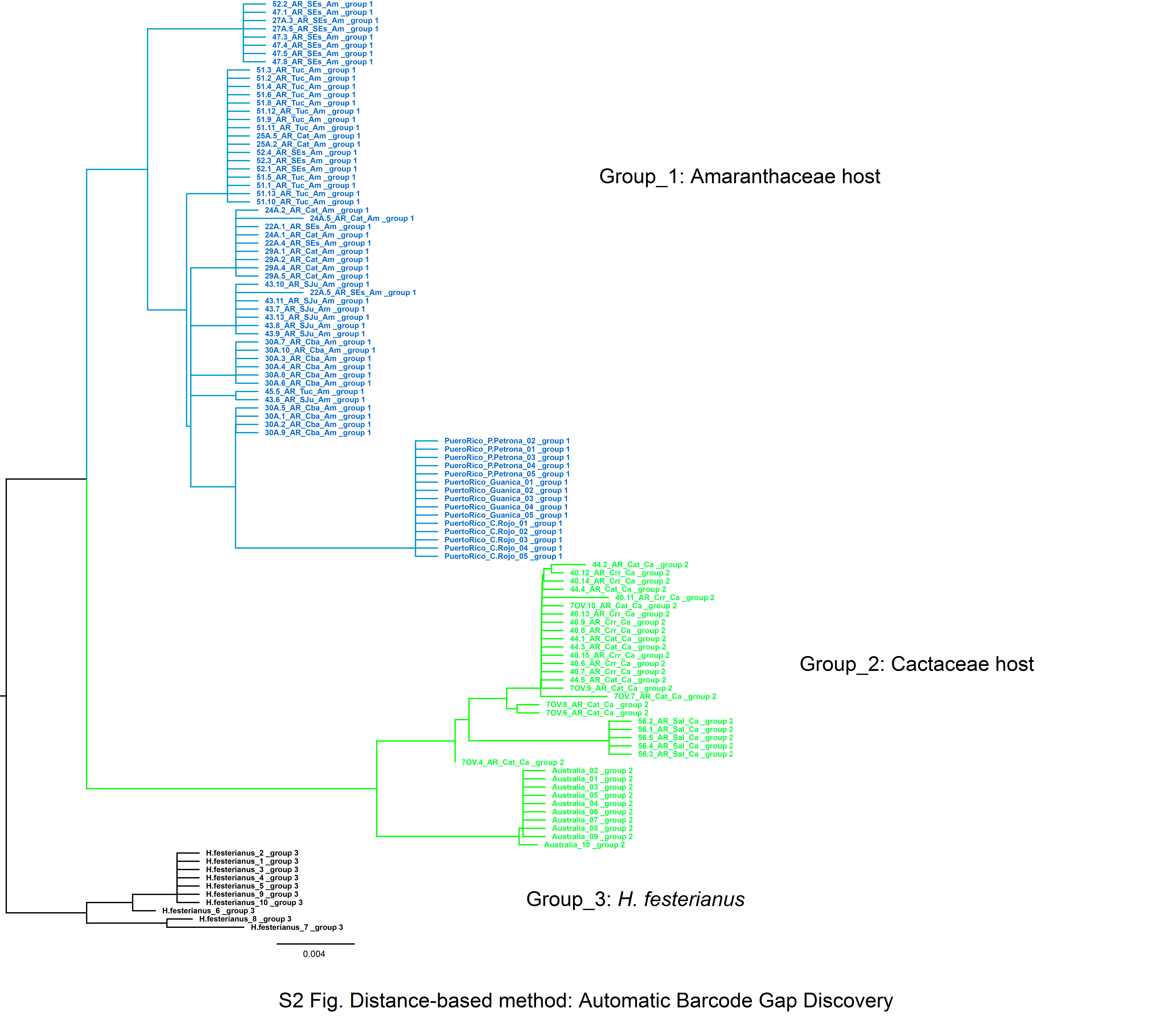

Supplement: S2 Fig — (TIF) [file pone.0220366.s002.tif]

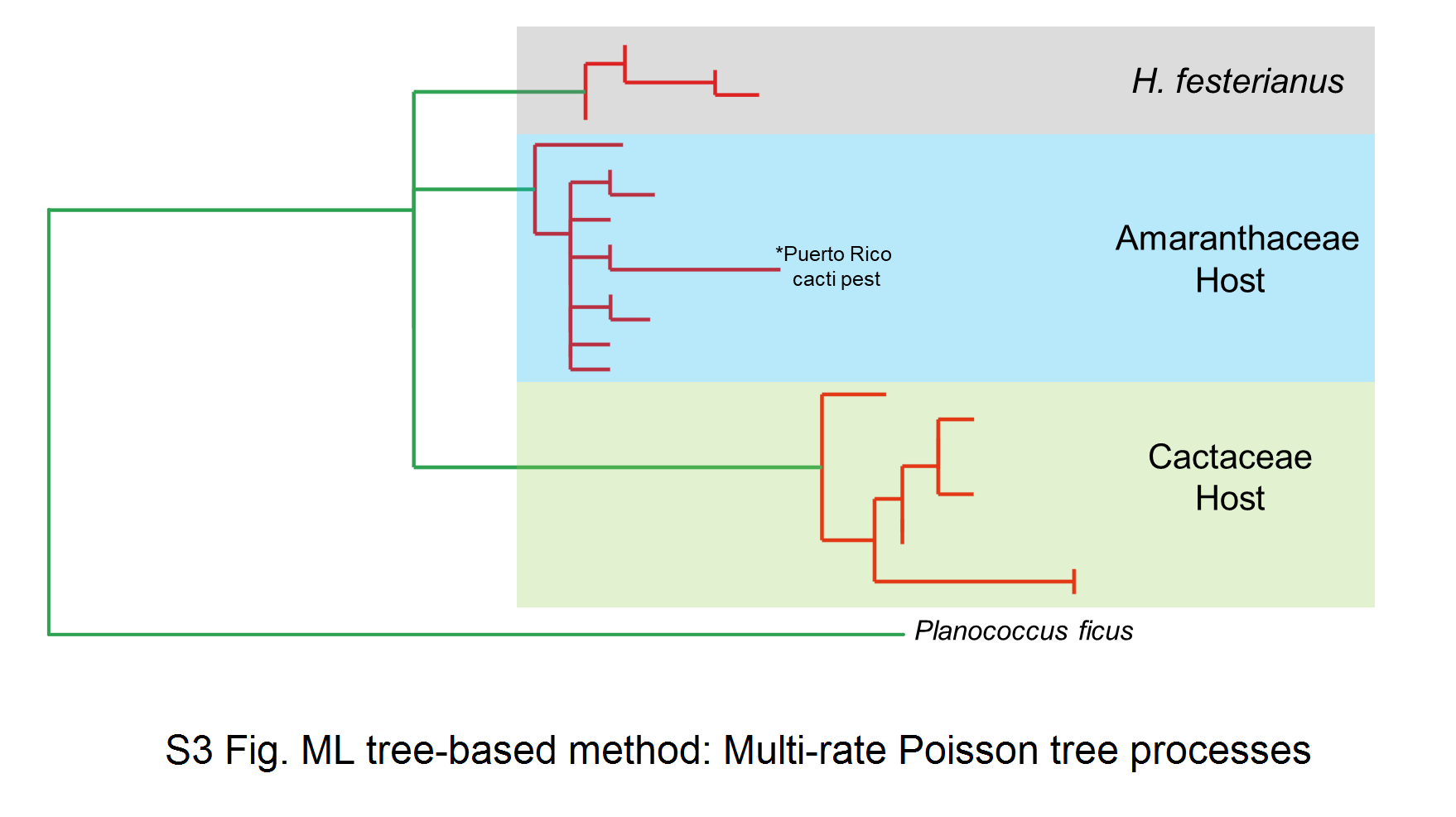

Supplement: S3 Fig — (TIF) [file pone.0220366.s003.tif]

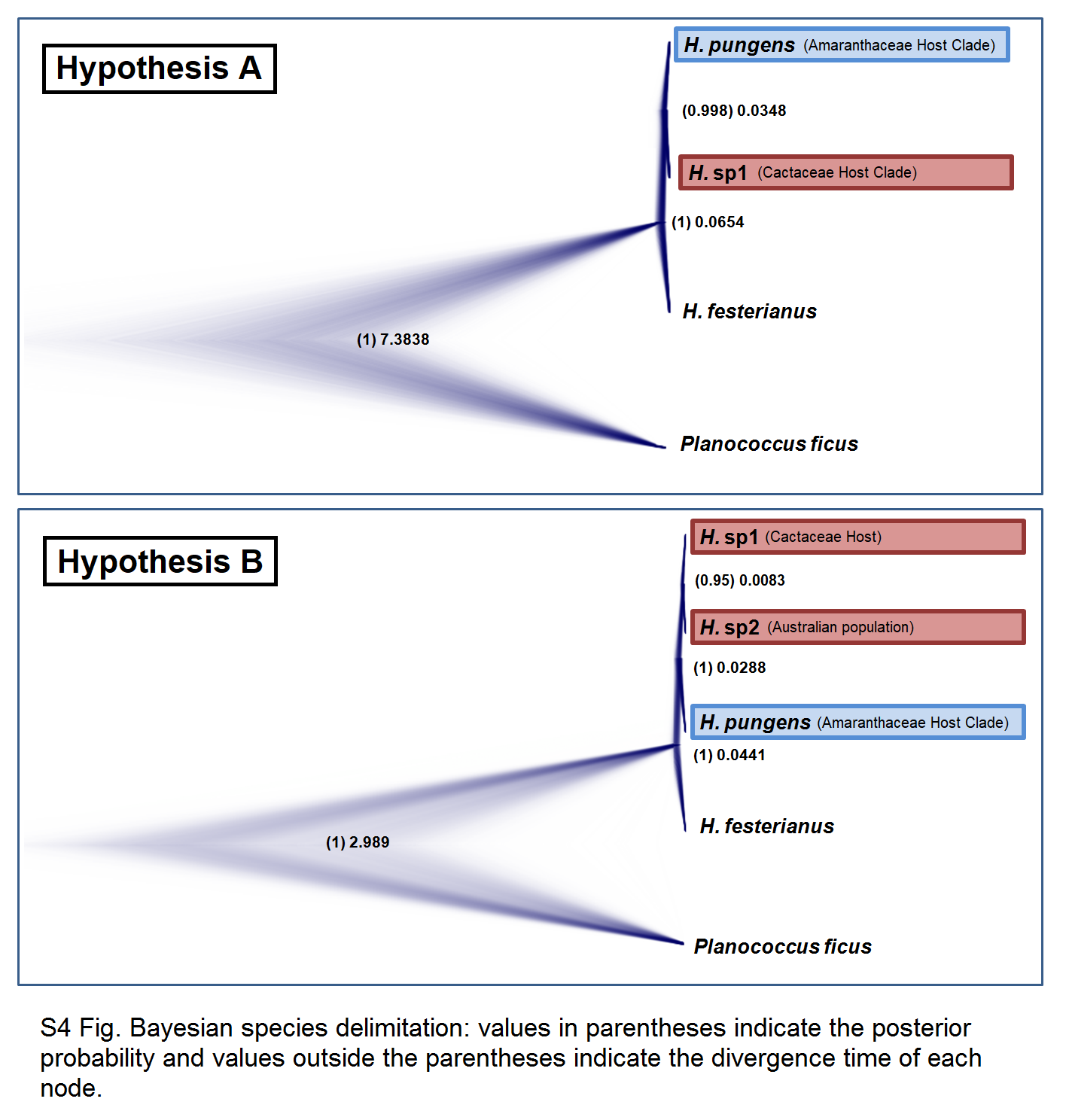

Supplement: S4 Fig — (TIF) [file pone.0220366.s004.tif]
