# Supplementary material for: Untangling the Hypogeococcus pungens species complex (Hemiptera: Pseudococcidae) for Argentina, Australia, and Puerto Rico based on host plant associations and genetic evidence
Source: PLoS One. 2019 Jul 25;14(7):e0220366. doi: 10.1371/journal.pone.0220366 (PMC6657911; doi:10.1371/journal.pone.0220366)
Supplement: S1 Table — (DOCX) [file pone.0220366.s005.docx]

**S1 Table. Geographical origin of the individuals analyzed**

| Country | Site | Family | Host plant | Latitude | Longitude | N° ind. |
| --- | --- | --- | --- | --- | --- | --- |
| *Hypogeococcus pungens* | | | | | | |
| Argentina | Corrientes | Cactaceae | *Cleistocactus baumannii* | -28.908444 | -58.661194 | 9 |
| Argentina | La Rioja | Amaranthaceae | *Gomphrena* sp. | -31.369778 | -66.868556 | 7 |
| Argentina | Catamarca | Cactaceae | *Cleistocactus baumannii* | -28.449528 | -65.633167 | 5 |
| Argentina | Tucumán | Amaranthaceae | *Alternanthera pungens* | -26.236722 | -65.274000 | 1 |
| Argentina | Santiago del Estero | Amaranthaceae | *Alternanthera pungens* | -28.627139 | -65.122214 | 5 |
| Argentina | Tucumán | Amaranthaceae | *Alternanthera pungens* | -27.769278 | -65.584528 | 12 |
| Argentina | Santiago del Estero | Amaranthaceae | *Alternanthera pungens* | -25.608086 | -65.646500 | 4 |
| Argentina | Salta | Cactaceae | *Cleistocactus baumannii* | -25.608086 | -65.646500 | 5 |
| Argentina | Mendoza | Cactaceae | *Cereus aethiops* | -34.64582 | -68.37605 | 3 |
| Argentina | Santiago del Estero | Amaranthaceae | *Alternanthera pungens* | -28.709333 | -65.108528 | 3 |
| Argentina | Catamarca | Amaranthaceae | *Alternanthera pungens* | -28.578639 | -65.633167 | 3 |
| Argentina | Catamarca | Amaranthaceae | *Alternanthera pungens* | -29.222389 | -65.773972 | 2 |
| Argentina | Santiago del Estero | Amaranthaceae | *Alternanthera pungens* | -28.146500 | -65.121611 | 2 |
| Argentina | Catamarca | Amaranthaceae | *Alternanthera pungens* | -29.605556 | -65.528417 | 4 |
| Argentina | Córdoba | Amaranthaceae | *Alternanthera pungens* | -30.973944 | -64.084056 | 10 |
| Argentina | Catamarca | Cactaceae | *Cleistocactus* sp. | -28.449528 | -65.633167 | 6 |
| Australia | Queensland | Cactaceae | *Harrisia martinii* | -27.597500 | 151.775556 | 10 |
| Puerto Rico | Cabo Rojo | Cactaceae | *Pilosocereus royenii* | 17.978800 | -67.169030 | 5 |
| Puerto Rico | Punta Petrona | Cactaceae | *Hylocereus trigonus* | 17.952231 | -66.383320 | 5 |
| Puerto Rico | Guanica | Cactaceae | *Melocactus intortus* | 17.959113 | -66.861479 | 5 |
| *Hypogeococcus festerianus* | | | | | | |
| Argentina | Mendoza | Cactaceae | *Cereus aethiops* | -32.864389 | -68.961639 | 3 |
| Argentina | Mendoza | Cactaceae | *Cereus aethiops* | -32.707440 | -68.856100 | 7 |
