# Supplementary material for: Untangling the Hypogeococcus pungens species complex (Hemiptera: Pseudococcidae) for Argentina, Australia, and Puerto Rico based on host plant associations and genetic evidence
Source: PLoS One. 2019 Jul 25;14(7):e0220366. doi: 10.1371/journal.pone.0220366 (PMC6657911; doi:10.1371/journal.pone.0220366)
Supplement: S2 Table — (DOCX) [file pone.0220366.s006.DOCX]

S2 Table. Evolutionary model, Substitution Saturation index and the sequence variation estimated for each gene evaluated in Jmodeltest v.2.1.3 (Darriba et al. 2012), DAMBE v.5.5.1 (Xia & Xie, 2001) and DnaSP v5 (Librado and Rosas 2009) respectively. Saturation substitution index (Iss), saturation substitution index critical (Iss.c), number of segregating sites (S), and number of haplotypes (H).

| **Molecular Marker** | **Evolutionary model** | | **Iss < or > Iss.c** | **S** | **H** |
| --- | --- | --- | --- | --- | --- |
| *COI* (432 bp) | GTR+Γ+I | 0,195 < 0,698 | | 52 | 21 |
| *18S* (548 bp) | GTR+Γ+I | 0,037 < 0,805 | | 24 | 9 |
| *EF1*α (435 bp) | GTR+Γ+I | 0,106 < 0,703 | | 66 | 4 |
